# Effect of DJ-1 on the neuroprotection of astrocytes subjected to cerebral ischemia/reperfusion injury

**DOI:** 10.1007/s00109-018-1719-5

**Published:** 2018-11-30

**Authors:** Li Peng, Yipeng Zhao, Yixin Li, Yang Zhou, Linyu Li, Shipeng Lei, Shanshan Yu, Yong Zhao

**Affiliations:** 1 Department of Pathology, Chongqing Medical University, Yixueyuan Road 1, 400016 Chongqing, People’s Republic of China; 2 Molecular Medical Laboratory, Chongqing Medical University, 400016 Chongqing, People’s Republic of China; 3 Institute of Neuroscience, Chongqing Medical University, 400016 Chongqing, People’s Republic of China; 4 Key Laboratory of Neurobiology, Chongqing Medical University, 400016 Chongqing, People’s Republic of China; 5 Department of Respiratory Medicine, Jiangjin Center Hospital, Chongqing, China

**Keywords:** Astrocyte, Co-culture, DJ-1, Nrf2/ARE, GSH, I/R injury

## Abstract

**Abstract:**

Astrocytes are involved in neuroprotection, and DJ-1 is an important antioxidant protein that is abundantly expressed in reactive astrocytes. However, the role of DJ-1 in astrocytes’ neuroprotection in cerebral ischemia/reperfusion injury and its potential mechanism is unclear. Thus, to explore effects and mechanisms of DJ-1 on the neuroprotection of astrocytes, we used primary co-cultures of neurons and astrocytes under oxygen and glucose deprivation/reoxygenation in vitro and transient middle cerebral artery occlusion/reperfusion in vivo to mimic ischemic reperfusion insult. Lentiviral was used to inhibit and upregulate DJ-1 expression in astrocytes, and DJ-1 siRNA blocked DJ-1 expression in rats. Inhibiting DJ-1 expression led to decreases in neuronal viability. DJ-1 knockdown also attenuated total and nuclear Nrf2 and glutathione (GSH) levels in vitro and vivo. Similarly, loss of DJ-1 decreased Nrf2/ARE-binding activity and expression of Nrf2/ARE pathway-driven genes. Overexpression of DJ-1 yielded opposite results. This suggests that the mechanism of action of DJ-1 in astrocyte-mediated neuroprotection may involve regulation of the Nrf2/ARE pathway to increase GSH after cerebral ischemia/reperfusion injury. Thus, DJ-1 may be a new therapeutic target for treating ischemia/reperfusion injury.

**Key Messages:**

- Astrocytes protect neurons in co-culture after OGD/R
- DJ-1 is upregulated in astrocytes and plays an important physiological roles in neuronal protection under ischemic conditions
- DJ-1 protects neuron by the Nrf2/ARE pathway which upregulates GSH

## Introduction

During cerebral ischemia/reperfusion (I/R) injury, oxidative stress is thought to be an important mediator of pathogenesis [1]. Neurons are susceptible under ischemic condition while astrocytes support neuronal function and survival during stress [2, 3]. Previous studies have shown that astrocytes protect neurons against oxidative stress and affect neuronal survival in some neurodegenerative diseases [2, 4]. In addition, neurons co-cultured with astrocytes are less sensitive to oxidative stress than when cultured alone [4, 5]. Thus, studying astrocyte-neuron interactions and identifying astrocyte-derived neuroprotective molecules may help treat cerebral ischemia/reperfusion injury.

DJ-1 is a multifunctional protein that regulates transcription [6, 7], antioxidant stress [8, 9], and anti-apoptotic processes [6, 10]. It is abundantly expressed in reactive astrocytes during chronic neurodegenerative diseases such as Parkinson’s disease [11, 12]. DJ-1 is also intensely expressed in immunoreactive astrocytes during ischemic stroke [13, 14] and important in regulating brain damage following ischemia [15]. However, the role of DJ-1 in astrocytes’ neuroprotection in cerebral ischemia/reperfusion injury and its potential mechanism is unclear. DJ-1 regulates Nrf2 and its downstream pathways, which are master transcription factors for oxidative stress [7, 16, 17]. In addition, there is an association between DJ-1 and glutathione (GSH) [18, 19].

GSH is an important antioxidant in the brain that participates in interactions between astrocytes and neurons [20–22]. Previous studies have shown that GSH is synthesized and released by astrocytes and can then be used by neurons to resist oxidative stress [21, 23]. In addition, elevated DJ-1 expression increases GSH levels in co-cultures of astrocytes and neurons [24], but it is not clear how these two molecules interact. DJ-1 may increase GSH by upregulating GSH synthesis to protect against oxidative stress during Parkinson’s disease [19]. Glutamate cysteine ligase (GCL), which includes the glutamate-cysteine ligase catalytic subunit (GCLC) and the glutamate-cysteine ligase regulatory subunit (GCLM), and glutathione synthesis (GSS) are necessary for synthesis of GSH [21, 25].

To determine whether the mechanism of action of DJ-1 in astrocyte-mediated neuroprotection involves GSH and Nrf2, we studied effects of lentivirus-mediated DJ-1 overexpression and knockdown on GSH and Nrf2 in experiments using oxygen and glucose deprivation/reoxygenation (OGD/R) and middle cerebral artery occlusion/reperfusion (MCAO/R) to mimic ischemic/reperfusion insult in vitro and in vivo, respectively.

## Materials and methods

### Animals and reagents

Astrocytes were prepared from postnatal 1-day-old Sprague-Dawley rats, and neurons were obtained from embryonic day 15–16 Sprague-Dawley rats. All experiments were authorized by the Institutional Animal Ethics Committee of Chongqing Medical University, Chongqing, China, and procedures were in compliance with the National Institutes of Health Guide for the Care and Use of Laboratory Animals. All efforts were made to minimize animal suffering.

Neurobasal medium, high-glucose Dulbecco’s Modified Eagle’s Medium/F12 (DMEM/F12), glucose-free Dulbecco’s Modified Eagle’s Medium (DMEM), B27, and fetal bovine serum (FBS) were obtained from Gibco (Grand Island, NY, USA). Hank’s solution and trypsin were purchased from HyClone (Logan, UT, USA). Poly-L-lysine was obtained from Sigma-Aldrich (Milan, Italy). Phosphate-buffered solution (PBS) and penicillin/streptomycin (Pen/Strep) were purchased from Beyotime (Shanghai, China). Lactate dehydrogenase (LDH), GSH, and γ-GCL assay kits were obtained from Jiancheng Bioengineering Institute (Nanjing, China). A CCK-8 kit was purchased from Dojindo (Kumamoto, Japan).

### MCAO/R and groups

Transient middle cerebral artery occlusion (MCAO)-reperfusion was performed and cerebral blood flow (CBF) was monitored as described previously [26–28]. Briefly, adult male Sprague-Dawley rats (250–280 g) were anesthetized with 3.5% chloral hydrate (350 mg/kg), and then placed on a heating pad to maintain body temperature at 37 ± 0.5 °C. A monofilament nylon suture (Shadong Biotechnology Company, Beijing, China) was inserted into the left middle cerebral artery. A laser Doppler flow meter (PeriFlux System 5000, Perimed, Sweden) was applied to detect CBF. The local CBF decreased to < 23.88 ± 2.08% of the baseline level after the middle cerebral artery was occluded. Then, the plug was removed after 1 h of ischemia to allow reperfusion and CBF was restored to ≥ 75% of baseline. After 4 h, 8 h, 12 h, and 48 h of reperfusion, brain tissue from the left cerebral cortex was assessed using Western blot analysis. After reperfusion 24 h, brain tissue was subjected to TTC, Western blot analysis, and immunohistochemistry. Sham animals were subjected to the same surgical procedure but without occlusion of the middle cerebral artery.

Adult male Sprague-Dawley rats were randomized to the following groups: (1) control group, (2) MCAO group, (3) negative control (NC) group: rats transfected with negative control siRNA (non-targeting sequence) and treated with MCAO, and (4) DJ-1 siRNA group: rats transfected with DJ-1 siRNA and treated with MCAO.

### DJ-1 interference in rats

The DJ-1 siRNA was designed and synthesized by Shanghai GenePharma Co., Ltd. (sense primer 5-CCCAUUGGCUAAGGACAAATT-3) and (antisense primer 5-UUUGUCCUUAGCCAAUGGGTT-3). As a control, a negative control siRNA without any target sequence was constructed (sense primer 5-UUCUCCGAACGUGUCACGUTT-3 and antisense primer5-ACGUGACACGUUCGGAGAATT-3). Twenty-four to forty-eight hours before MCAO, DJ-1siRNAs were injected into the left lateral cerebral ventricle. Western blot analysis was used to confirm knockdown efficiency of DJ-1siRNA.

### Cell cultures

Astrocyte-rich primary cultures were established using the method of Park and colleagues [29]. Briefly, after maintenance of astrocyte cultures for 2–3 weeks, microglia and oligodendrocytes were dislodged and removed from flasks with mild shaking. The remaining astrocytes were detached by trypsinization, and digested cells were plated directly on the plastic surface in 24-well plates. When astrocytes reached 80% confluence, neuron-enriched cultures were prepared using previously published methods [26]. Neurons were plated onto poly-L-lysine-coated, PBS pre-equilibrated, 14-mm glass coverslips in 24-well plates. Neuron purity (> 90%) was assessed using the neuron-specific marker, NeuN.

**Fig. 1:**
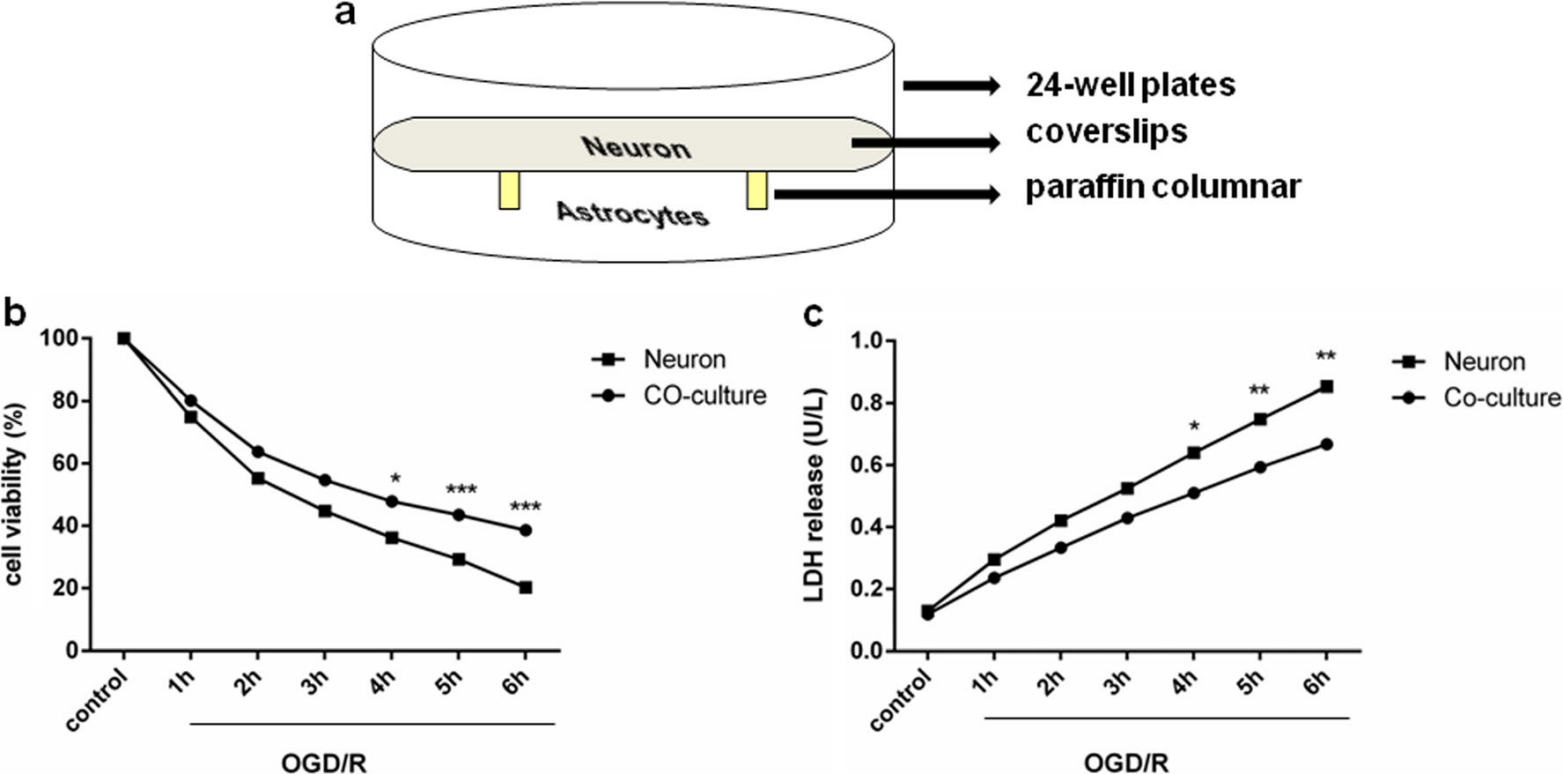
Co-culture with astrocytes increased neuronal survival after OGD/R. **a** Non-contact neuron-astrocyte co-culture system. **b** Cell viability data. *n* = 6 of samples/group from an experiment, three independent experiments were carried out. **c** LDH release. *n* = 6 of samples/group from an experiment, three independent experiments were carried out. OGD/R = oxygen-glucose deprivation/reoxygenation. Values are expressed as mean ± SEM. Neuronal monoculture vs. co-culture; *p < 0.05, **p < 0.01, ***p < 0.001

Non-contact neuron-astrocyte co-cultures were established as previously described with modifications [30]. The co-culture model is shown in Fig. 1a. Briefly, when neurons were almost adhered to a poly-L-lysine-coated coverslips on paraffin column without glial cells in culture medium containing 2% B27 and 1% Pen/Strep, they were incubated with astrocyte monolayers in a manner that did not allow physical contact between the two cell types. The paraffin column was not injurious to cultures.

### OGD/R and groups

OGD/R was performed as previously described. The co-culture system was established with glucose-free DMEM in an incubator with 94% N_2_, 1% O_2_, and 5% CO_2_ for 1, 2, 3, 4, 5, and 6 h at 37 °C. Then, the medium was replaced with normal culture medium (neurobasal medium with 2% B27 and 1% Pen/Strep) in an incubator with 5% CO_2_ and 95% air for 24 h at 37 °C.

Co-cultures were randomized to the following groups: (1) control: untreated cells, (2) OGD/R treatment, (3) negative control (NC): astrocytes transfected with negative control lentivirus (non-targeting sequence lentivirus) and treated with OGD/R, (4) knockdown: astrocytes transfected with DJ-1 knockdown lentivirus and treated with OGD/R, (5) scramble: astrocytes transfected with scramble lentivirus and treated with OGD/R, and (6) overexpression: astrocytes transfected with DJ-1 overexpressing lentivirus and treated with OGD/R.

### Lentiviral transfection of shRNA

Lentivirus was obtained from Neuron Biotech (Shanghai, China). The following three RNA sequences based on the rat DJ-1 gene (Gene ID: NM_117287) were used: 5-CCGGACGGCAGTCACTACAGCTACTCAAGAGATAGCTGTAGTGACTGCCGTTTTTTTG-3 (knockdown), 5-CCGGTTCTCCGAACGTGTCACGTTTCAAGAGAACGTGACACGTTCGGAGAATTTTTTG-3 (NC: It was the control sequence of the knockdown sequence), and 5-ATGGACTACAAGGATGACGATGACAAGGATTACAAAGACGACGATGATAAGGACTATAAGGATGATGACGACAAA-3 (overexpression). Scramble lentivirus did not contain inserts. Astrocytes were transfected with knockdown or NC at a multiplicity of infection (MOI) of 10 in medium and overexpression or scramble at a MOI of 15 in medium. Green fluorescent protein (GFP)-positive (overexpression and scramble) cells and red fluorescent protein (RFP)-positive (knockdown and NC) cells were confirmed under a fluorescence microscope. Sustained DJ-1 downregulation or overexpression efficiency was confirmed by Western blot analysis and quantitative real-time PCR (q-PCR).

### q-PCR arrays

Differences in mRNA expression between groups were assessed using q-PCR as previously reported (Carrión et al. 2014; Lu et al. 2012). Total RNA from astrocytes was extracted using RNAiso Plus (TaKaRa Biotechnology, Dalian, China), and RNA was converted to complementary DNA (cDNA) by reverse transcription (TaKaRa Biotechnology). Then, q-PCR was performed using TaKaRa SYBR Premix Ex Taq II (Tli RNase H Plus) (TaKaRa Biotechnology) with a CFX96 Touch™ Real-Time PCR Detection System. Probe and primer sequences (Sangon Biotech, Shanghai, China) were as follows for DJ-1: forward, 5′-AGGAGCAGAGGAGATGGAGA-3′, reverse, 5′-ACATCACGGCTACACTGCAC-3′.

### Western blots

Astrocyte protein lysates and brain tissue protein lysates were prepared in RIPA buffer containing protease inhibitor PMSF. Equal amounts of protein (50 μg per lane) were resolved with SDS-PAGE and electrotransferred onto PVDF membranes (Millipore, Boston, MA, USA). Membranes were then blocked with 5% non-fat milk TBST buffer at room temperature and incubated at 4 °C overnight with the following primary antibodies: anti-DJ-1 (1:2000, Abcam, Cambridge, UK, ab76008), anti-Nrf2 (1:200, Santa Cruz Biotechnology, CA, USA, SC-722), anti-CRIF1 (1:500, Proteintech, Chicago, IL, USA, 16260-1-AP), anti-GCLC (1:500, Proteintech, 12601-1-AP), anti-GCLM (1:500, Proteintech, 14241-1-AP), anti-GSS (1:500, Proteintech, 15712-1-AP), and anti-β-actin (1:5000, ABclonal, Wuhan, China, AC004). The next day, membranes were washed and incubated with HRP-conjugated secondary antibodies (1:5000, ABclonal, AS014, AS003) for 2 h at room temperature. Protein bands were visualized with an ECL kit (Millipore, Temecula, CA, USA). Band intensity was quantified using ImageJ and normalized to intensity of the β-actin band. Three independent experiments were performed.

### Immunocytochemistry and immunohistochemistry

Cells grown on glass coverslips and frozen sections of brain tissue were lightly fixed in 4% paraformaldehyde, blocked with 3% FBS/0.01% Triton X-100 in PBS, and incubated at 4 °C overnight with the following primary antibodies: anti-DJ-1 (1:50, Abcam, ab76008), anti-Nrf2 (1:50, Abcam, ab31163), and anti-GFAP (1:200, ABclonal, A10871). The next day, cells or sections were incubated with secondary antibodies at room temperature. Secondary antibodies were tagged with either DyLight 488 (anti-mouse, Abbkine, green, A23210) at 1:200 or DyLight 549 (anti-rabbit, Abbkine, red, A23320) at 1:200. Coverslips and sections were mounted with Vectashield containing DAPI. Sections and cells were imaged with a fluorescence microscope or a laser scanning confocal microscope.

### Assessment of infarct volume

After 24 h of reperfusion, brains were sliced into 2-mm-thick sections and stained with 2% 2,3,5-triphenyltetrazolium chloride (TTC, Sigma, USA) at 37 °C for 15 min in the dark, and then fixed in 4% paraformaldehyde at 4 °C for 24 h. Each section was photographed with a digital camera, and areas of volume were analyzed using an ImageJ (version 6.0, NIH, Bethesda, MD, USA). Percentage of infarct volume was calculated using the following equation: {[total infarct volume − (ipsilateral hemisphere volume − contralateral hemisphere volume)] / contralateral hemisphere volume} × 100%.

### Assessment of neurological deficit scores and brain water content

After 24 h of reperfusion, neurologic deficit scores were assessed by an observer blinded to the experimental animals using a modified scoring system in accordance with the methods previously reported [27]. The scoring system was as follows: grade 0, no neurological deficits; grade 1, unable to entirely extend contralateral forelimb; grade 2, circling to the right; grade 3, falling to the contralateral side; grade 4, no spontaneous autonomic activity or loss of consciousness; grade 5, dead.

Rats were killed 24 h after reperfusion and the brains were rapidly removed and weighed to obtain the wet weight. After 24 h of drying in an oven at 120 °C until the weight was invariable, the brains were weighed again to obtain the dry weight. Brain water content was calculated as follows: (wet weight − dried weight) / wet weight × 100%.

### Electrophoretic mobility shift assay

Electrophoretic mobility shift assay was conducted using a commercial kit (Gel Shift Assay System; Promega, Madison, WI, USA). The Nrf2 consensus oligonucleotide probe (5′-TGG GGA ACC TGT GCT GAG TCA CTG GAG-3′) was end-labeled with [c-32P] ATP (Sangon Biotech., Shanghai, China) using T4-polynucleotide kinase. Then, 15 μg of nuclear protein was incubated in a binding buffer for 15 min at room temperature. Samples were then loaded onto a non-denaturing 4% polyacrylamide gel and electrophoretically separated in 0.25× TBE buffer. Gels were vacuum-dried and exposed to X-ray film (Fuji Hyperfilm, Tokyo, Japan) at 80 °C with an intensifying screen. Nrf2 DNA binding activity was quantified with computer-assisted densitometric analysis.

### LDH assay

After OGD/R, cell culture medium was collected from each group. LDH released into the culture medium was measured using an assay kit (Jiancheng Bioengineering Institute, Nanjing, China) according to the manufacturer’s instructions. Absorbance was read at 440 nm using a microplate reader (Bio-Rad, Foster City, CA, USA), and LDH activity was expressed as U/L.

### Cell viability

Neuronal viability was assessed using a CCK-8 kit (Dojindo, Kumamoto, Japan) according to the manufacturer’s protocol. Briefly, after cells were subjected to OGD/R, 100 μl CCK-8 was added to each well, and samples were incubated at 37 °C for up to 2 h. Absorbance was read at 450 nm, and cell viability was expressed as relative percent compared with controls.

### ELISA measurement of GSH

To investigate the level of GSH expression in rats, the GSH amount was measured using GSH enzyme-linked immunosorbent assay (ELISA) kit (Mbbiology Institute, JiangSu, China) according to the manufacturer’s instructions. And absorbance was read at 450 nm.

### GSH and γ-GCL assays

Cell culture medium was collected from each group. GSH and γ-GCL activity was assayed in culture medium using kits (Jiancheng Bioengineering Institute, Nanjing, China) according to the manufacturer’s instructions. Data were collected at 405 and 660 nm.

### Statistical analysis

Data are expressed as mean ± SEM. All statistical analyses were performed using GraphPad Prism software (version 6.0). Statistical differences among multiple groups were determined by one-way analysis of variance (ANOVA) followed by the Tukey’s test for multiple comparisons. Statistical significance was established when *P* < 0.05.

## Results

### Co-culture with astrocytes increased neuronal survival after OGD/R

Monocultured neurons and co-culture system were subjected to OGD for different time periods ranging from 1 to 6 h; after 24 h of reoxygenation, cell viability was tested by CCK-8 kit. Figure 1b shows that neuronal viability gradually decreased after extended OGD/R. Co-cultured neurons exhibited greater survival compared with monocultured neurons. LDH activity (Fig. 1c**)** increased with increasing OGD/R time, and it was lower in co-cultures compared with neuronal monocultures. Thus, astrocytes protect neurons against OGD/R.

### DJ-1 ameliorated cerebral (I/R) injury after MCAO/R

**Fig. 2:**
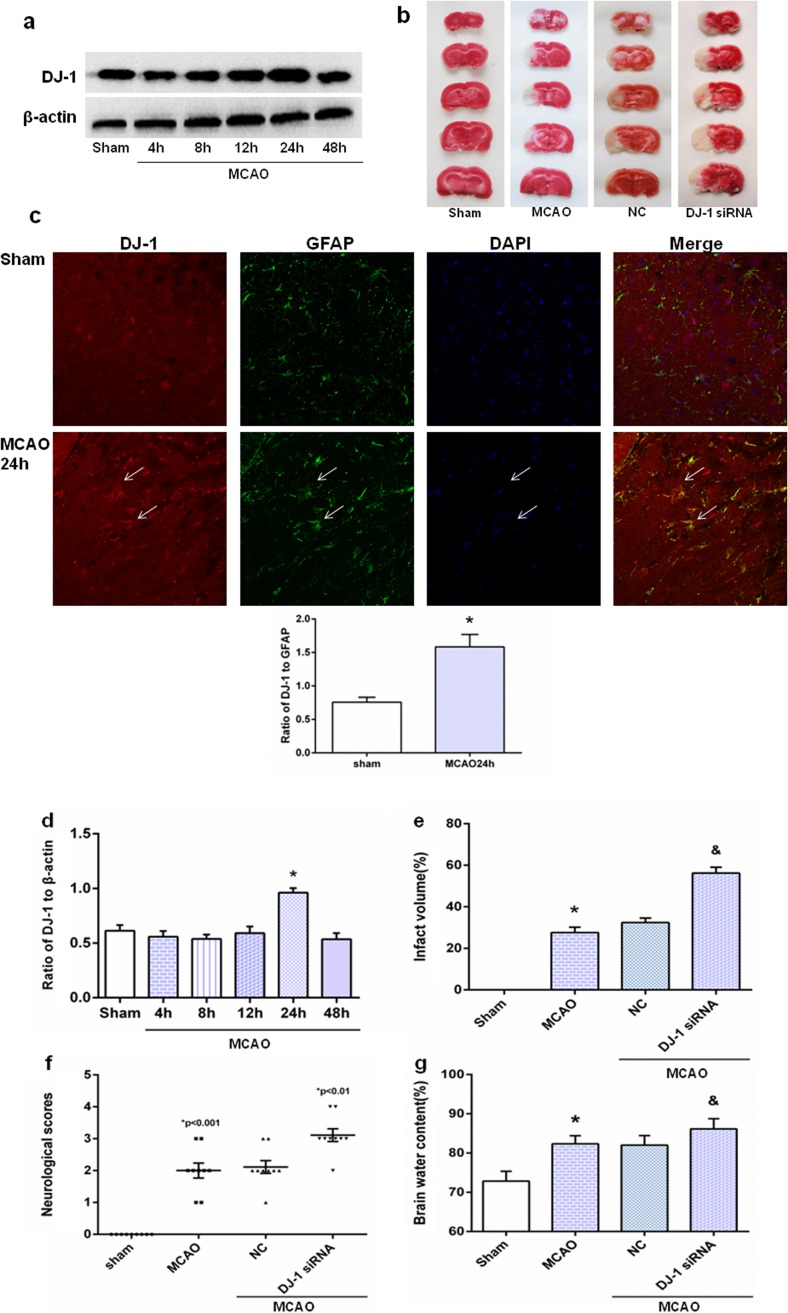
DJ-1 ameliorated cerebral (I/R) injury after MCAO/R. **a** Western blot of DJ-1. **d** Ratios of DJ-1 relative to β-actin. In the 24-h group, DJ-1 levels increased compared with the sham group. Values are mean ± SEM. *p < 0.05. *n* = 3 of samples/group from an experiment, three independent experiments were carried out. **c** Immunohistochemistry for DJ-1 and semi-quantitation to determine percentage of DJ-1-postive cells relative to GFAP-positive cells in astrocytes. In the 24-h MCAO group, DJ-1 was expressed at higher levels in reactive astrocytes in cortical infarct regions compared with controls. Laser scanning confocal microscope was used to assess expression. DJ-1 expression in astrocytes is indicated by red fluorescence. GFAP expression in astrocytes is indicated by green fluorescence. Cell nuclei were stained with DAPI. Original magnification, 400×. Values are expressed as mean ± SEM. *p < 0.05 vs. sham. *n* = 3 of samples/group from an experiment, three independent experiments were carried out. **b**, **e** Infarct volume of brain. **f** Neurological scores. **g** Brain water content. Infarct volume, neurological scores, brain water content were significantly increased in the DJ-1 siRNA group compared with the MCAO and NC groups. Values are mean ± SEM. *p < 0.01 vs. sham; &*p* < 0.05 vs. MCAO, *n* = 3 of samples/group from an experiment, three independent experiments were carried out

We analyzed the changes of DJ-1 in ischemic rats with increasing reperfusion time (Fig. 2a, d) and showed higher levels of DJ-1 at reperfusion 24 h when compared with the sham group, and there were no differences among the other treatment groups. Double staining of brain sections with antibodies against GFAP and DJ-1 showed that reactive astrocytes expressed DJ-1 protein in cortical infarct regions in the 24-h reperfusion group (Fig. 2c). To explore the effects of DJ-1 on cerebral I/R injury, cerebral infarct volume, neurological deficit scores, and brain water content were used to detect the effects of DJ-1 on cerebral I/R injury. Figure 2b, e shows that infarct volume was significantly increased in the DJ-1 siRNA group compared with the MCAO group, and there were no differences in infarct volume between the MCAO and NC groups. Similarly, silencing DJ-1 could remarkably increase neurological deficit scores and brain water content compared with the MCAO group (Fig. 2f, g). These results indicate that DJ-1 can ameliorate cerebral I/R injury.

### DJ-1 expression in astrocytes varied with OGD duration

**Fig. 3:**
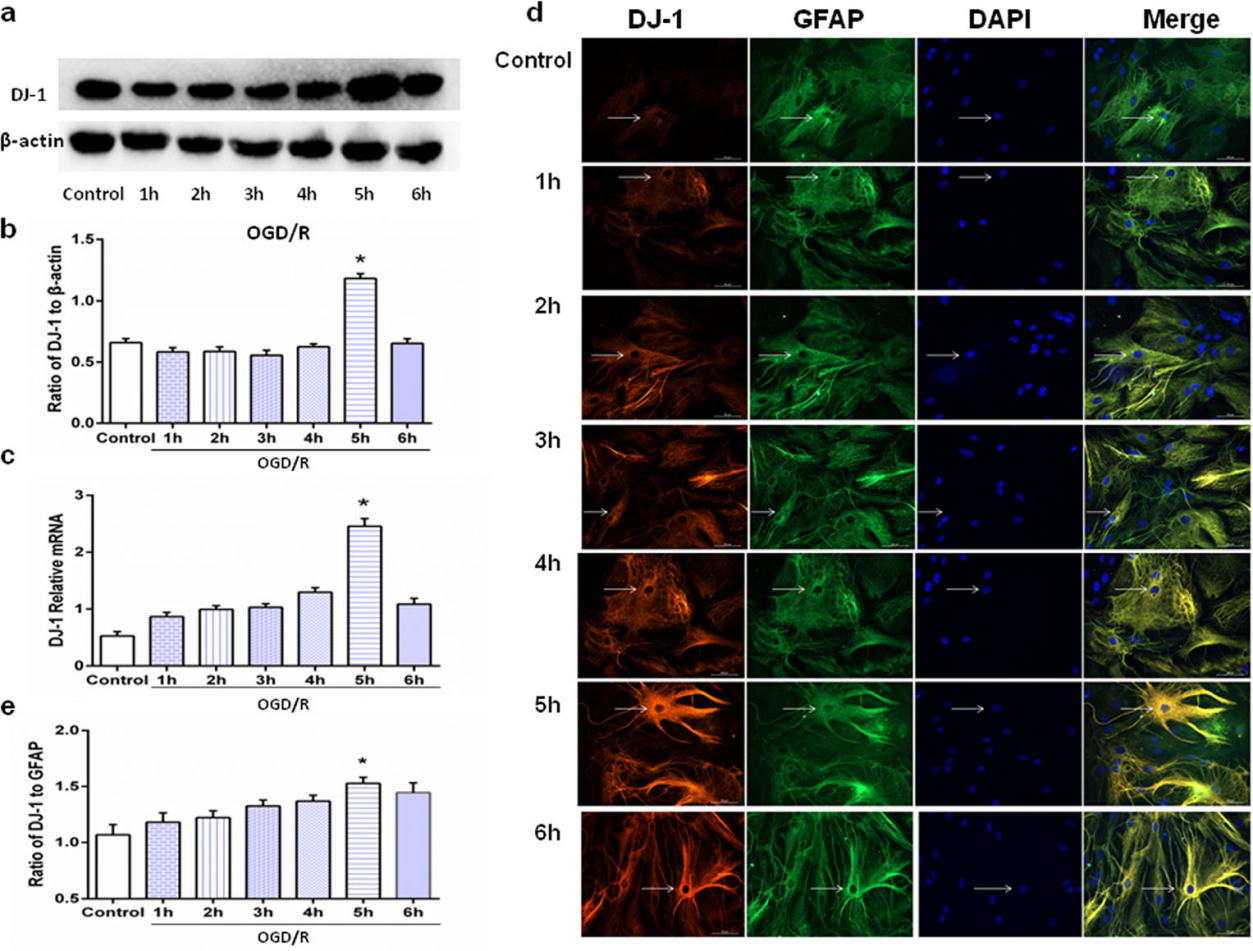
DJ-1 expression in astrocytes varied with OGD duration. After OGD/R, astrocytes were harvested. **a** Western blot analysis of DJ-1. **b** Ratios of DJ-1 relative to β-actin showing that DJ-1 in the 5-h group increased compared with controls. **c** q-PCR data was consistent with results of Western blots. **d** Immunocytochemistry to measure DJ-1 expression in astrocytes. Fluorescence microscopy was used to assess expression. DJ-1 expression in astrocytes is indicated by red fluorescence. GFAP expression in astrocytes is indicated by green fluorescence. Cell nuclei were stained with DAPI. Original magnification, 200×. **e** Semi-quantitation to determine percentage of DJ-1-postive cells relative to GFAP-positive cells in astrocytes. Values are mean ± SEM. *p < 0.05, *n* = 3 of samples/group from an experiment, three independent experiments were carried out

To test whether astrocytic DJ-1 levels increased after OGD/R treatment, we used Western blot and q-PCR to detect expression levels of DJ-1 protein and mRNA, respectively. Figure 3a–c shows that DJ-1 was upregulated in the 5-h OGD group compared with the controls, and there were no differences in DJ-1 expression in the remaining treatment groups. Immunocytochemistry (Fig. 3d, e) confirmed that expression of DJ-1 was higher in the 5-h group compared with the control group. These results suggest that DJ-1 expression is upregulated when co-cultured cells are subjected to OGD/R, and the maximal effect was observed in the 5-h group. Therefore, to explore neuroprotective effects of DJ-1 and its mechanisms of action, we chose OGD 5 h as the optimum time point for further study.

### Astrocytic DJ-1 protected neurons by upregulating GSH levels

**Fig. 4:**
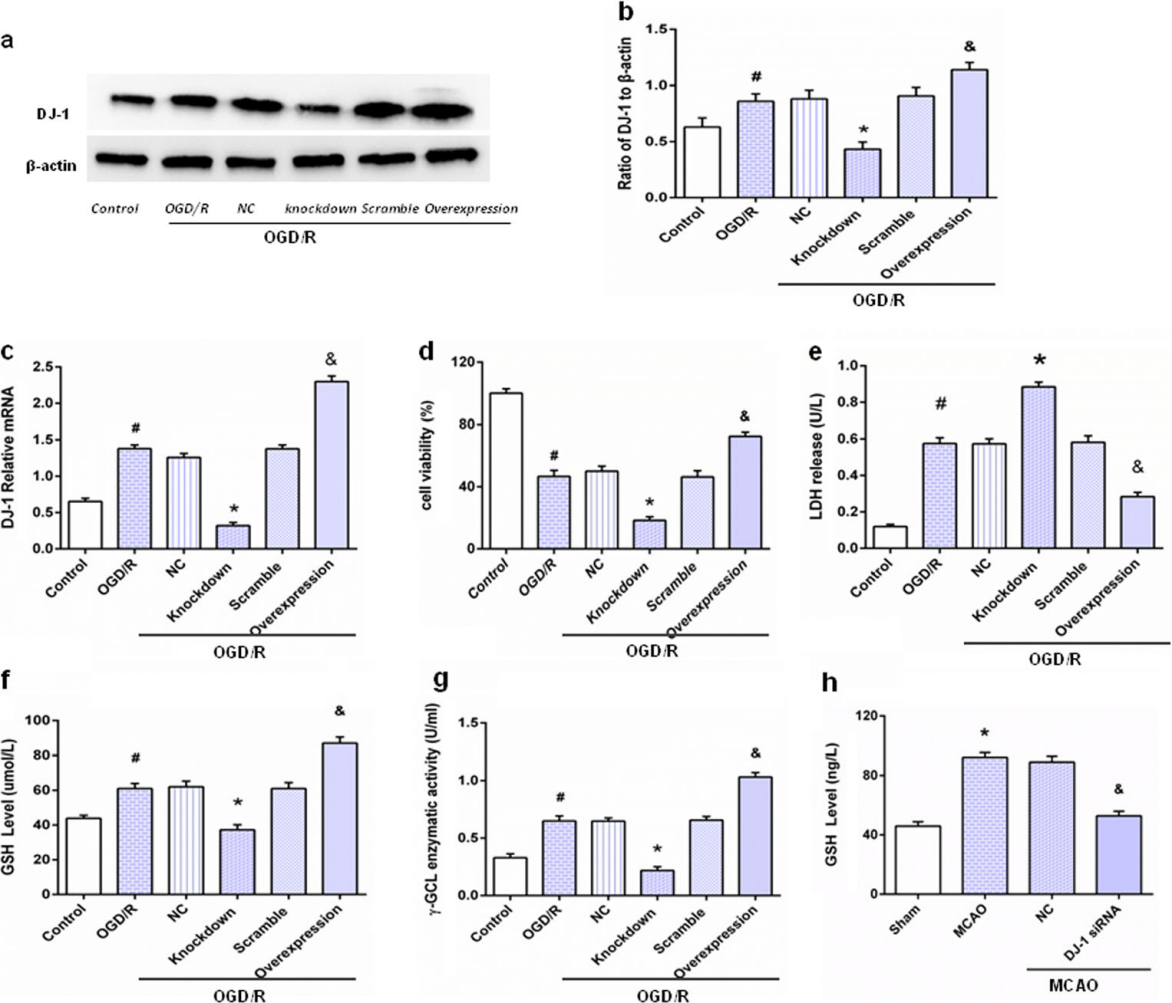
Astrocytic DJ-1 protected neurons by upregulating GSH levels. After OGD for 5 h followed by reoxygenation for 24 h, astrocytes, neurons, and culture medium were harvested. **a** Western blot of DJ-1. **b** Ratios of DJ-1 relative to β-actin. DJ-1 expression was reduced in the knockdown group and upregulated in the overexpression group. **c** q-PCR results were consistent with Western blots. Values are expressed as mean ± SEM. #*p* < 0.05 vs. control; *p < 0.01 vs. OGD/R; &*p* < 0.01 vs. OGD/R. *n* = 3 of samples/group from an experiment, three independent experiments were carried out. **d** Neuronal viability after exposure to OGD/R. **e** LDH in the culture medium. **f** GSH in the culture medium. **g** γ-GCL in the culture medium. **h** GSH in rats. Values represent mean ± SEM. #*p* < 0.05 vs. control; *p < 0.01 vs. OGD/R; &*p* < 0.05 vs. OGD/R. *n* = 6 of samples/group from an experiment, three independent experiments were carried out. Values are mean ± SEM. *p < 0.01 vs. sham; &*p* < 0.01 vs. MCAO, *n* = 3 of samples/group from an experiment, three independent experiments were carried out

Knockdown lentivirus and overexpressing lentivirus were continuously applied from 72 h to knockdown and overexpress the DJ-1. Western blotting and qPCR were used to detect the knockdown and overexpression efficiency. Western blot analysis (Fig. 4a, b) showed that DJ-1 expression decreased after lentiviral knockdown and increased after lentiviral overexpression when compared with the OGD/R group, and these data are consistent with q-PCR results in Fig. 4c.

The CCK-8 kit was used to test the effects of DJ-1 on neuron viability after exposure to OGD/R. As shown in Fig. 4d, compared with the OGD/R treatment group, cells in the knockdown group subjected to lentiviral-mediated interference of DJ-1 were less viable, and viability was increased in the DJ-1 overexpression group. Silencing DJ-1 increased LDH activity (Fig. 4e), and overexpression decreased it. To investigate the neuroprotective mechanisms of DJ-1, GSH level and GCL activity in the culture medium was measured. Figure 4f, g shows that overexpressing DJ-1 increased GSH levels and GCL activity compared with the OGD/R group. DJ-1 knockdown had the opposite effect. Similarly, GSH levels in rats were measured. Figure 4h shows that loss of DJ-1 decreased GSH levels.

### Effects of DJ-1 on expression of Nrf2

**Fig. 5:**
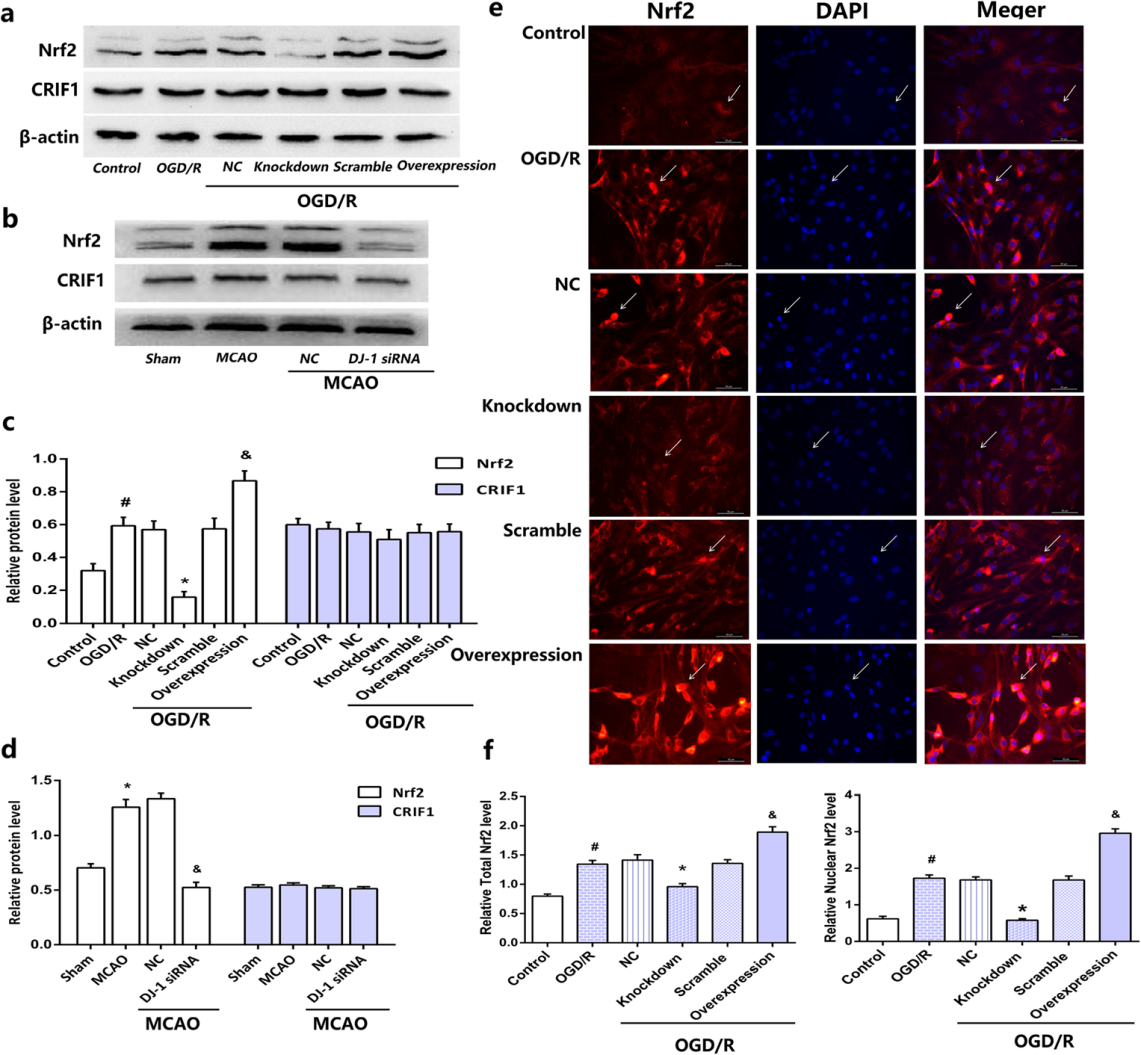
Effects of DJ-1 on expression of Nrf2. **a** After OGD for 5 h followed by 24 h of reoxygenation, astrocytes were harvested. Western blot for Nrf2 and CRIF1 in astrocytes. **b** After 24 h of reperfusion, brains were collected. Western blot for Nrf2 and CRIF1 in rats. **c**, **d** Ratios of Nrf2 and CRIF1 relative to β-actin in astrocytes and in rats, respectively. **e** Immunocytochemistry assays to measure total Nrf2 and nuclear Nrf2 in astrocytes. Fluorescence microscopy was used to assess expression. Nrf2 expression in astrocytes is indicated by red fluorescence. Cell nuclei were stained with DAPI. Original magnification, 200×. **f** Semi-quantitation to determine total number of Nrf2-positive cells and number of nuclear Nrf2-positive cells in astrocytes. Values are mean ± SEM. #*p* < 0.05 vs. control; *p < 0.05 vs. OGD/R; and &*p* < 0.05 vs. OGD/R. *n* = 3 of samples/group from an experiment, three independent experiments were carried out. Values are mean ± SEM. *p < 0.01 vs. sham; &*p* < 0.01 vs. MCAO, *n* = 3 of samples/group from an experiment, three independent experiments were carried out

Western blotting was used to detect the expression of total Nrf2 and CRIF1. After OGD/R, expression of total Nrf2 increased compared with controls, and after DJ-1 knockdown, expression of total Nrf2 decreased compared with the OGD/R group. Overexpression of DJ-1 had the opposite effect (Fig. 5a, c). Similarly, DJ-1 interference decreased levels of total Nrf2 compared with the MCAO group in rats (Fig. 5b, d). However, changes in DJ-1 expression had no effect on levels of CRIF1 in cells and rats (Fig. 5a–d). Results of immunocytochemistry experiments presented in Fig. 5e, f confirmed that expression of total Nrf2 and nuclear Nrf2 in astrocytes significantly increased in the OGD/R group compared with the controls. Silencing DJ-1 decreased Nrf2 expression, and overexpressing DJ-1 increased Nrf2 expression. No changes were observed in the other treatment groups.

### DJ-1 regulated Nrf2/ARE binding activity and Nrf2/ARE-driven gene expression

**Fig. 6:**
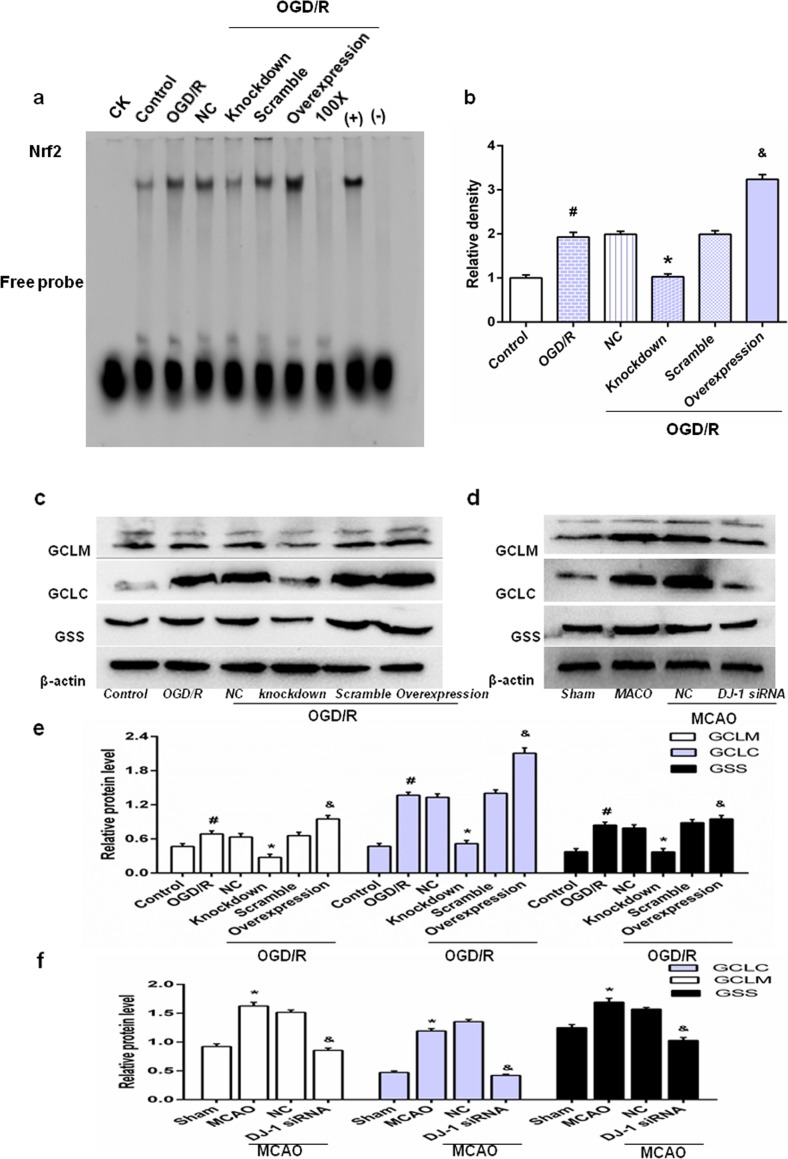
DJ-1 regulates Nrf2/ARE binding activity and Nrf2/ARE-driven gene expression. After OGD for 5 h followed by 24 h of reoxygenation, astrocytes were harvested. And after 24 h of reperfusion, brains were collected. **a** EMSA analysis of Nrf2/ARE binding. **b** Semiquantitative analysis of Nrf2/ARE binding. CK, 100x, (+) and (−) indicate controls. **c** Western blot for GCLM, GCLC, and GSS in astrocytes. **d** Western blot for GCLM, GCLC, and GSS in rats. **e**, **f** Ratios of GCLM, GCLC, and GSS relative to β-actin in astrocytes and in rats, respectively. Values are mean ± SEM. #*p* < 0.01 vs. control; *p < 0.01 vs. OGD/R; and *p* < 0.05 vs. OGD/R. *n* = 3 of samples/group from an experiment, three independent experiments were carried out. Values are mean ± SEM. *p < 0.05 vs. sham; &*p* < 0.01 vs. MCAO, *n* = 3 of samples/group from an experiment, three independent experiments were carried out

Astrocyte nuclear extracts were subjected to EMSA for measurement of Nrf2-ARE binding activity. The data (Fig. 6a, b) indicated no differences among the OGD/R, NC, and scramble groups. Compared with the OGD/R group, treatment with DJ-1 interfering lentivirus reduced Nrf2 binding activity, and the opposite effect was observed in the overexpression group (*p* < 0.05). Thus, after OGD/R, DJ-1 may increase Nrf2/ARE binding.

Western blot analysis was used to investigate the effects of DJ-1 on expression of the Nrf2/ARE-driven genes, GCLC and GCLM and GSS. The data (Fig. 6c, e) showed that GCLC, GCLM, and GSS expression did not differ between the OGD/R, NC, and scramble groups. After knockdown of DJ-1, expression of GCLC, GCLM, and GSS decreased compared with the OGD/R group. After overexpression of DJ-1, expression of GCLC, GCLM, and GSS increased compared with the OGD/R group. Similarly, Fig. 6d, f shows that silencing DJ-1 interference decreased expression of GCLC, GCLM, and GSS compared with the MCAO group. Thus, DJ-1 upregulates expression of Nrf2/ARE-driven genes GCLC, GCLM, and GSS.

## Discussion

We explored mechanisms underlying action**s** of DJ-1 in astrocytes and noted that DJ-1 was upregulated in astrocytes under ischemic conditions. After knockdown and overexpression of DJ-1, neuronal survival decreased or increased, respectively. Loss of DJ-1 decreased GSH levels and Nrf2 expression in vitro and vivo; overexpression of DJ-1 had the opposite effect. Similarly, the Nrf2/ARE binding and expression of antioxidant proteins GCLC, GCLM, and GSS that act downstream of ARE followed trends in DJ-1 expression. Thus, astrocytic DJ-1 mediates neuroprotection by regulating the Nrf2/ARE pathway to upregulate GSH.

Astrocytes protect neurons against chronic impairment of oxidative metabolism [4, 5]. Long-term survival of cultured neurons is improved when they are co-cultured with glial cells [5, 21]. Consistent with these results, we found that viability of neurons co-cultured with astrocytes was greater than neurons cultured alone. We used neurobasal medium to support the greater nutritional requirements for the co-cultured neurons [28, 30].

DJ-1 is an antioxidant protein expressed in neurons and glial cells [31, 32]. However, expression of DJ-1 is increased in reactive astrocytes in sporadic Parkinson’s disease and other neurodegenerative diseases [12, 14]. Previous work demonstrated that DJ-1 is increased in immunoreactive astrocytes 24 h after reperfusion following stroke [13], and we noted similar results at all reperfusion time points in a time-dependent manner [13]. DJ-1 significantly increased 24 h after reperfusion in reactive astrocytes in vivo. In vitro, we found that DJ-1 peaked at 5 h of OGD/R in astrocytes. This may be an antioxidant stress response or a protective response. In this study, DJ-1 overexpression increased neuronal viability. DJ-1 participates in astrocyte-mediated neuroprotection [33] by scavenging reactive oxygen species (ROS) or inducing astrocyte synthesis and release of protective molecules [24, 34].

GSH is a soluble factor that is synthesized and released from astrocytes and participates in astrocyte-mediated neuroprotection [21, 25]. DJ-1 increases GSH and reduces neuronal damage induced by oxidative stress [24]. In contrast, Mullett and colleagues showed that DJ-1 did not affect GSH after cell treatment with rotenone [35]. We noted that DJ-1 was neuroprotective through GSH release by astrocytes after OGD/R. This may be the result of a different model. GSH can scavenge cellular ROS as an enzyme or in a non-enzymatic manner [36, 37]. Cysteine in GSH is released by astrocytes and used to create neuronal GSH [38, 39]. And lactate from astrocytes is provided to neurons for ATP production [40]. Cysteine from astrocyte is a major substrate for neuronal de novo GSH biosynthesis. The biosynthesis process via two stepwise ATP-dependent reactions and the two rate-limiting steps were regulated by GCL and GS, respectively [21].

In studies to determine how DJ-1 regulates GSH, we observed that knockdown of DJ-1 decreased expression of Nrf2 and prevented nuclear accumulation of Nrf2. Overexpression of DJ-1 yielded the opposite effects in astrocytes. Consistent with previous studies, we found that DJ-1 mediates Nrf2 activation and eventual nuclear translocation of Nrf2 in astrocytes [7, 16]. However, in non-brain cells, DJ-1 stabilizes Nrf2 by inhibiting its ubiquitination and promotes Nrf2 nuclear translocation by preventing its binding with Keap1 [7, 41]. Other studies showed that DJ-1 inhibits CRIF1-mediated Nrf2 degradation to regulate Nrf2 protein stability and Nrf2 translocation to the nucleus [42, 43]. We found that CRIF1 levels did not change with alterations in DJ-1 expression. Perhaps different cells have unique sensitivities to oxidative stress. Nrf2 is a key transcription factor that binds to ARE to antagonize injury induced by oxidative stress [44, 45]. Thus, EMSA was used to test Nrf2/ARE binding, and we noted similar changes with DJ-1. Nrf2 is often the central signaling switch that regulates expression of phase II detoxifying enzymes and antioxidants, such as GCLC, GCLM, and GSS, through the Nrf2/ARE pathway [45–48]. Nrf2 downstream proteins, GCLC, GCLM, and GSS, decreased after DJ-1 interference, but overexpression of DJ-1 upregulated expression of GCLC, GCLM, and GSS in vitro.

**Fig. 7:**
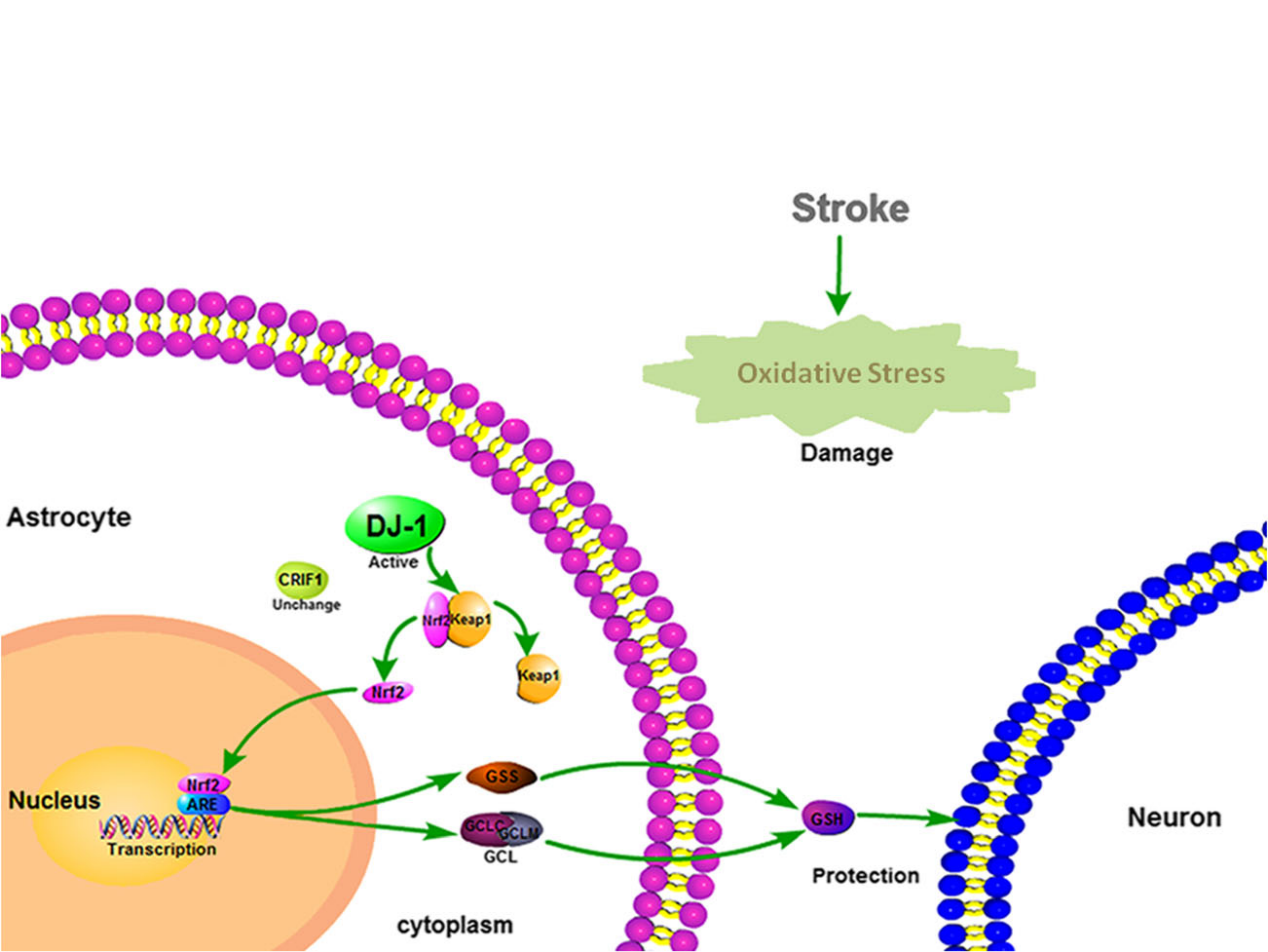
Mechanisms of action of DJ-1 in astrocytes. After oxidative stress induced by cerebral ischemia and reperfusion, DJ-1 is expressed in immunoreactive astrocytes. DJ-1 facilitates Nrf2 translocation to the nucleus by preventing binding with Keap1. Nrf2 binds to ARE and upregulates expression of GCLC, GCLM, and GSS, which regulate synthesis of GSH

In conclusion, DJ-1 may play important roles in neuronal protection, and these effects may be mediated by the Nrf2/ARE pathway, which upregulates GSH in vitro*.* The mechanism is shown in the Fig. 7. This suggests a novel mechanism of action for astrocytic DJ-1 in OGD/R and that DJ-1 may provide antioxidant activity after stroke.
